# Clinical Predictors of Engagement in Teleintegrated Care and Telereferral Care for Complex Psychiatric Disorders in Primary Care: a Randomized Trial

**DOI:** 10.1007/s11606-021-07343-x

**Published:** 2022-02-02

**Authors:** Jennifer Severe, Paul N. Pfeiffer, Katherine Palm-Cruz, Theresa Hoeft, Rebecca Sripada, Matthew Hawrilenko, Shiyu Chen, John Fortney

**Affiliations:** 1 Department of Psychiatry, University of Michigan Medical School, 4250 Plymouth Rd, Ann Arbor, MI 48109 USA; 2 Department of Veterans Affairs, Ann Arbor Veterans Affairs Center for Clinical Management Research, Ann Arbor, MI USA; 3 Department of Psychiatry and Behavioral Sciences, School of Medicine, University of Washington, Seattle, WA USA; 4 Department of Veterans Affairs, Health Services Research and Development, Center of Innovation for Veteran-Centered and Value-Driven Care, Seattle, WA USA

**Keywords:** telehealth, collaborative care, treatment engagement, psychiatric disorders, PTSD, bipolar disorder, primary care, federally qualified, health centers

## Abstract

**Background:**

Telepsychiatry Collaborative Care (TCC) and Telepsychiatry/Telepsychology Enhanced Referral (TER) expand the reach of specialty mental health services to underserved populations.

**Objective:**

Assess clinical predictors of treatment engagement for complex psychiatric conditions in TCC—in which remote specialists consult with primary care teams via an onsite care manager who also provides brief psychotherapy—and TER, in which remote specialists provide direct telehealth treatment.

**Design:**

A randomized pragmatic trial from twenty-four primary care clinics without onsite psychiatrists or psychologists.

**Participants:**

A total of 1,004 adult patients screened positive for posttraumatic stress disorder (PTSD)and/or bipolar disorder were randomized to receive TCC or TER for 1 year.

**Main Measures:**

Psychotherapy engagement was measured by the number of sessions completed, and pharmacotherapy engagement by the medication adherence item from the Schizophrenia Care and Assessment Program Health Questionnaire (SCAP-HQ).

**Key Results:**

Engagement in TCC psychotherapy visits was greater compared to TER. There was no association between the PTSD symptom severity and treatment engagement. The internal state scale (ISS) activation subscale, an indicator of mania, was associated with reduced odds of initiating psychotherapy (odds ratio [OR] = 0.70; 95% CI, 0.59 to 0.84) but not the number of sessions attended once psychotherapy started. The Drug Abuse Screening Test-10(DAST-10) score was associated with receipt of fewer psychotherapy sessions (incidence ratio rate [IRR] = 0.88; 95% CI, 0.81 to 0.95). The number of physical health comorbidities was associated with greater engagement in psychotherapy (IRR = 1.11, 95% CI, 1.03 to 1.19) and pharmacotherapy (OR = 1.54; 95% CI, 1.27 to 1.87). None of the findings varied by intervention group.

**Conclusions:**

Both teleintegrated and telereferral care offer an opportunity to treat patients with complex psychiatric conditions. While there was no difference in clinical characteristics predicting engagement, onsite care managers engaged patients in more psychotherapy sessions than remote therapists.

**Trial Registration:**

ClinicalTrials.gov Identifier: NCT02738944

**Supplementary Information:**

The online version contains supplementary material available at 10.1007/s11606-021-07343-x.

Due to limited availability of mental health specialists in underserved areas, primary care practices often treat patients with complex psychiatric conditions^1–3^, including posttraumatic stress disorder (PTSD) and bipolar disorder (BD) which respectively affects up to 20%^4,5^ and 4%^3^ of their population. Bipolar disorder and PTSD frequently co-occur^6^ and have a high likelihood of psychiatric comorbidities^7–9^, including substance use disorders^10,11^, resulting in greater symptom burden and lower quality of life^6,8^, premature mortality^9,12^, and higher disengagement from care^10,13–15^. A review of empirically supported treatments for PTSD yielded attrition rates as high as 60% for veterans^16^ and 57% for the general population^17^. Premature dropout rates for pharmacotherapy for BD range between 30 and 40%^13,18^.

Telepsychiatry Collaborative Care (TCC) and Telepsychiatry/Telepsychology Enhanced Referral (TER) expand the reach of specialty mental health services to underserved populations^19–22^. TCC uses a team-based approach with a lead primary care provider, support from a care manager and consultation from a telepsychiatrist for treatment recommendations^21,22^. TER allows for live synchronous audio-visual interactions between patients and mental health clinicians for delivery of care^20,21^. Telepsychiatrists prescribed medications directly to the patient and telepsychologists deliver psychotherapy. Both models are evidence-based practices for depressive and anxiety disorders^20,21^ but little is known regarding the engagement of patients with complex psychiatric conditions. Prior studies showed PTSD severity and the symptom cluster of avoidance to be associated with patient dropout in specialty mental health care ^23–25^. Studies of patients with BD demonstrated a switch to hypomania negatively impacts treatment engagement, particularly when associated with substance use^8,18^. Whether PTSD and BD symptomatology and comorbidities are associated with engagement in TCC or TER, or differences in engagement between models, could inform individual patient care decisions, treatment guidelines, and service implementation for millions of Americans increasingly relying on telehealth services^26^.

We assessed clinical predictors of psychotherapy and pharmacotherapy engagement using data from the *Study to Promote Innovation in Rural Integrated Telepsychiatry* (SPIRIT), a large comparative effectiveness trial of TCC (an integrated care model), and TER (a virtually co-located, but not integrated, model), for the management of PTSD and BD in underserved primary care settings^27^. Improved outcomes were observed in both intervention groups, and there was overall greater engagement in psychotherapy in the TCC arm^28^. This secondary analysis focuses on potential differences in clinical predictors of engagement in treatment across and between arms to inform patient-level clinical decision-making. We hypothesized that PTSD *avoidance* symptoms would be associated with decreased engagement across both arms. We also conducted exploratory analyses to assess whether overall PTSD symptoms, mania, and clinical covariates were associated with treatment engagement and whether the effects differed by care model. We hypothesized that negative predictors of engagement would have stronger effects in the TER arm compared to the TCC arm, as the latter offers more proactive efforts to engage and activate patients through the primary care-based care manager.

## METHOD

### Study Design

**Figure 1:**
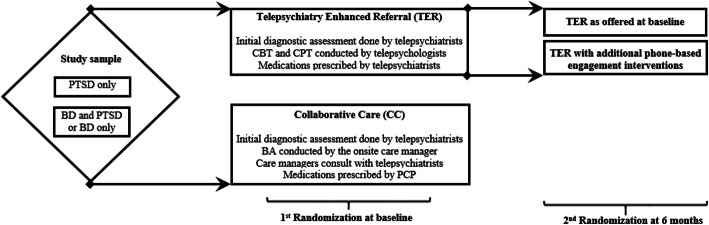
**SPIRIT Sequential Multiple Assignment Randomized Trials (SMART).**

SPIRIT is a randomized pragmatic comparative effectiveness trial (Fig. 1). Randomization to TCC or TER was stratified by screening results (PTSD-only group versus BD-only or BD and PTSD group). Virtually, all participants (92%) who screened positive for BD also screened positive for PTSD. If participants randomized to TER had not engaged in the first 6 months of the trial (having ≤ 2 interactive video encounters), they were randomized a second time to receive a phone call from a psychologist encouraging usage of the telehealth services (Fig. 1). The enrollment target was 1,000 patients.

### Study Settings and Participants

The SPIRIT trial was conducted in 24 clinics associated with 12 Federally Qualified Health Centers (FQHCs) across three states in the USA (Arkansas, Michigan, and Washington). FQHCs are community-based health centers that provide preventive and primary care services to rural, underserved areas and minority communities regardless of patients’ ability to pay^29^. FQHC clinics were recruited to participate if they had no psychiatrist or psychologist on site. The study protocol was approved and monitored by the Institutional Review Boards (IRB) at the participating state medical schools (University of Arkansas for Medical Sciences, University of Michigan, and University of Washington) and participating FQHCs entered into a reliance agreement to receive oversight from these IRBs. The SPIRIT trial was developed in close collaboration with our Consumer Advisory Board and Policy Advisory Board^27^.

Eligible patients were 18 years or older, not currently prescribed a psychotropic medication by a mental health specialist, English-speaking, and had positive screen on the abbreviated PTSD Checklist (PCL-6 ≥14) for PTSD^30^and/or on the Composite International Diagnostic Interview 3.0 (CIDI) (positive stem question responses and score of ≥8) for BD^31^. Patients with any physical and mental health comorbidities were included as well as patients with safety concerns according to a safety protocol put in place.

### Study Recruitment and Screening

The PCL-6 and CIDI were used for recruitment purposes only. To minimize screening burden, only patients who screened positive for depression on the Patient Health Questionnaire^32^ (PHQ-9 score ≥ 10) were subsequently screened by clinic staff for PTSD and BD to enter the study. A total of 2,464 patients screened positive for PTSD on the PCL-6and/or BD on the CIDI. Among those, 1,931 patients were found eligible and 1,214 consented to participate in the SPIRIT trial. A total of 1,004 participants completed the baseline research assessment and were randomized to an intervention group (TCC vs TER). Patients were recruited from November 16, 2016, to June 30, 2019.

### Study Interventions

TCC telepsychiatrists and TER telepsychiatrists and telepsychologists were offsite, university affiliated, and credentialled to practice at the FQHC. Both TCC and TER psychiatrists conducted an initial assessment through telehealth to assign clinical diagnoses and develop a treatment plan. In TER, the telepsychiatrists prescribed all psychotropic medications and provided referrals to telepsychologists. Telepsychologists delivered cognitive processing therapy (CPT) for PTSD, cognitive behavioral therapy (CBT) for BD, and either one or both for patients with both PTSD and BD. In the TCC arm, the onsite primary care physicians prescribed psychotropic medications recommended by the offsite telepsychiatrists who discussed individual cases with the onsite care managers. The care managers were registered nurses and licensed clinical social workers who were trained to deliver evidence-based behavioral activation (BA)^33^.

### Definition of Engagement

We measured medication engagement in the past 2 weeks by the medication adherence item from the Schizophrenia Care and Assessment Program Health Questionnaire (SCAP-HQ)^34^ administered during the 12-month follow-up survey. Those who responded that they never missed or missed only a couple times their medications on the SCAP-HQ item were considered engaged in pharmacotherapy. Those who refused a prescription or not taking their medications as prescribed were considered not engaged. We measured psychotherapy engagement by the number of visits completed with the offsite telepsychologists or onsite care managers.

### Clinical Predictors of Engagement

Psychometrically validated scales were administered to participants at baseline and 6 and 12 months later. This included the PTSD Checklist for DSM-5(PCL-5) for PTSD^4^; the 20-item Hopkins Symptom Checklist Depression Scale (SCL-20) for depression^35^; the Drug Abuse Screen Test (DAST-10) for drug use^36^; the Alcohol Use Disorders Identification Test (AUDIT-C) for alcohol misuse^37^; and the Depression Outcomes Module Comorbidity Checklist for physical comorbidities^38^. We used the Internal State Scale (ISS) for BD mood states. It comprises four subscales including the Activation subscale which identifies a manic or mixed state^39,40^.

### Data Analysis

Psychotherapy engagement was modeled with a zero-inflated negative binomial model. The zero-inflated model decomposes engagement into two related outcomes with separate parameter estimates: a logistic regression coefficient for the binary zero/nonzero process, which models whether patients initiated any psychotherapy, and a negative binomial coefficient for the distinct count process, which models the number of sessions they attended. Pharmacotherapy engagement was modeled as a binary outcome using a logistic regression.

We examined hypotheses stepwise using a series of nested models. We tested whether PTSD symptom severity was predictive of treatment engagement by modeling each respective outcome using the PCL-5 total score as the only predictor. To examine whether the effect of avoidance varied from other PTSD symptom domains, we used likelihood ratio tests to compare the model with the overall PCL-5 score to a model with separate cluster-specific scores. We included an interaction term to assess whether PTSD symptoms had differential effects by treatment arm. We then explored the predictive value of the additional clinical characteristics by adding these to the model. These series of nested models were all fitted to the same size of dataset for likelihood ratio tests, which is 981 out of 1004. Twenty-three cases with missing data were excluded. We used the Benjamini-Hochberg^41^ adjustment with the false discovery rate set to 5% to adjust for multiple comparisons.

## RESULTS

### Study Participants and Screening results

#### Participant Characteristics

A total of 1,004 participants were randomized to the TCC arm (*n*=506) and to the TER arm (*n*=498). In TER, 228 (46.0%) did not engage and were re-randomized to receive additional phone contacts. This adaptive component of the TER intervention was not effective and therefore was not factored into this analysis. Study participants had a mean age 39.6 years (SD 13.0) and 70% were female and 34% were racial/ethnic minorities. Only 31% were employed full- or part-time, and 62% lived below the 2016 Federal Poverty Level. Medicaid and Medicare beneficiaries accounted for 68% and 24% respectively. The sample had worse than average mental health-related quality of life as measured by the Veterans RAND 12-item Health Survey Mental Health Composite Score (MCS)^42^(Table 1).

**Table 1:** Baseline Characteristics of Patient Enrolled in SPIRIT Trial

	Full sample (*N*=1004) *N* (%) or *μ* (SD)	BPD (*N*=367) *N* (%) or *μ* (SD)	PTSD (*N*=637) *N* (%) or *μ* (SD)
**Age** (mean (SD))	39.40 (12.9)	37.59 (11.7)	40.44 (13.4)
**Gender** (%)			
Female	703 (70.0)	243 (66.2)	460 (72.2)
Male	285 (28.4)	117 (31.9)	168 (26.4)
Other gender identity	12 (1.2)	4 (1.1)	8 (1.3)
Missing	4 (0.4)	3 (0.8)	1 (0.2)
**Race** (%)			
African American	118 (11.8)	55 (15.0)	63 (9.9)
Hispanic Caucasian	660 (65.7)	231 (62.9)	429 (67.3)
Multi-race	63 (6.3)	31 (8.4)	32 (5.0)
Non-Hispanic Caucasian	77 (7.7)	22 (6.0)	55 (8.6)
Other	41 (4.1)	13 (3.5)	28 (4.4)
Missing	45 (4.5)	15 (4.1)	30 (4.7)
**Education** (%)			
College graduate or more	126 (12.5)	39 (10.6)	87 (13.7)
Some college	339 (33.8)	128 (34.9)	211 (33.1)
High school graduate	315 (31.4)	119 (32.4)	196 (30.8)
Some high school or less	222 (22.1)	80 (21.8)	142 (22.3)
Missing	2 (0.2)	1 (0.3)	1 (0.2)
**Employment** (%)			
Disabled	17 (1.7)	8 (2.2)	9 (1.4)
Retired	96 (9.6)	24 (6.5)	72 (11.3)
Student	33 (3.3)	11 (3.0)	22 (3.5)
Temporarily laid off or on strike	14 (1.4)	4 (1.1)	10 (1.6)
Unemployed	504 (50.2)	198 (54.0)	306 (48.0)
Work full-time	187 (18.6)	78 (21.3)	109 (17.1)
Work part-time	125 (12.5)	40 (10.9)	85 (13.3)
Missing	28 (2.8)	4 (1.1)	24 (3.8)
**Poverty** (%)			
Above	326 (32.5)	121 (33.0)	205 (32.2)
Below	620 (61.8)	231 (62.9)	389 (61.1)
Missing	58 (5.8)	15 (4.1)	43 (6.8)
**Uninsured**	71 (7.1)	21 (5.7)	50 (7.8)
**Psychotropic medication** (mean (SD))	24.66 (12.3)	23.18 (11.1)	25.49 (12.8)
**Mental Health Component**(MCS) score (range: 0–100)	30.84 (23.0)	29.50 (22.9)	31.62 (23.1)

#### Screening Results

About two-thirds of the study sample (*n*=637, 63%) screened positive for PTSD only and about one-third (*n*=367, 37%) screened positive for BD. Nearly all of those who screened positive for BD (92%) also screened positive for PTSD.

### Participant Clinical Characteristics

The mean score on the PCL-5 was 47.75 (SD 17.7) suggesting moderate PTSD severity and on the SCL-20 was 2.44 (SD 0.7) indicating that patients were moderately to quite a bit bothered by depressive symptoms. The mean ISS score was 179.44 (SD 121.0), with 53.5% meeting criteria for mania or a mixed state. The AUDIT-C showed no alcohol misuse for 80% of patients. The DAST-10 revealed on average a low to moderate level of problems related to drug abuse. The mean number of physical health comorbidities was 4 (SD 2.7) with high prevalence for repeated problems with neck, back, or spine (62%); migraine (46%); and arthritis or any kind of rheumatism (43%) (Supplementary Table 3).

### Psychotherapy Engagement

The number of psychotherapy encounters was 60% greater in the TCC intervention group compared to the TER group. Of the TCC patients, 79.3% had ≥1 BA encounter and averaged 9.6 encounters (SD=7.7). Of the TER patients, 45% had ≥1 telepsychology encounter and averaged 6.4 encounters (SD=4.4). The PCL-5 cluster-specific scores examined separately (intrusion, avoidance, negative alterations in cognitions and mood, and alterations in arousal and reactivity) did not have differential effects on psychotherapy engagement and did not improve the model fit compared to using the total PCL-5 score (*χ*^2^(6) = 7.65, *p* = .26; supplementary Table 4). Therefore, we used the PCL-5 total scores in subsequent models. Overall, the PCL-5 severity score was not significantly associated with psychotherapy initiation, or the overall number of sessions attended, and the association did not vary by intervention group (*χ*^2^(2) = 3.03, *p* = .21). Including additional clinical covariates, such as the SCL-20, ISS activation, DATS-10, AUDIT-C, and physical comorbidities, provided a significantly better fit to the data than excluding them (*χ*^2^(10) = 51.83, *p* < .001), but did not significantly impact the main findings. The ISS activation, indicative of manic or a mixed state, was associated with psychotherapy initiation but not the number of sessions attended once patients initiated treatment. A one standard deviation increase in the ISS activation subscale was associated with reduced odds (odds ratio = 0.70; 95% CI, 0.59 to 0.84) of engaging in psychotherapy. A one standard deviation increase in the DAST-10 score was associated with attending 12% fewer sessions (incidence rate ratio = 0.88; 95% CI, 0.81 to 0.95) and a one standard deviation increase in the number of physical comorbidities was associated with attending 11% more sessions (incidence rate ratio = 1.11; 95% CI, 1.03 to 1.19) (Table 2).

**Table 2:** Prediction Models for Psychotherapy Engagement and Pharmacotherapy Engagement

Predictor	Psychotherapy engagement (binary)		Psychotherapy engagement (count)		Pharmacotherapy engagement	
	Odds ratio (95% CI)	adj. *p*	Incidence rate ratio (95% CI)	adj. *p*	Odds ratio (95% CI)	adj. *p*
PCL-5 overall	1.18 (0.96, 1.45)	.20	1.04 (0.96, 1.14)	.44	0.8 (0.64, 1.01)	.16
SCL-20	1.17 (0.94, 1.44)	.20	0.98 (0.89, 1.08)	.72	1.11 (0.88, 1.39)	.51
ISS activation	0.7 (0.58, 0.83)	<.001	0.95 (0.88, 1.03)	.36	0.93 (0.77, 1.12)	.51
DAST-10	0.89 (0.75, 1.05)	.20	0.88 (0.81, 0.95)	.005	0.9 (0.74, 1.08)	.43
AUDIT-C	1.12 (0.93, 1.35)	.23	0.99 (0.92, 1.06)	.72	0.96 (0.79, 1.15)	.64
Physical comorbidities	1.17 (0.99, 1.38)	.13	1.1 (1.02, 1.18)	.036	1.54 (1.27, 1.87)	<.001

### Pharmacotherapy Engagement

Pharmacotherapy engagement increased over time, with no differences between intervention groups. The PCL-5 cluster-specific scores examined separately showed no differential associations with medication adherence (*χ*^2^(3) = 1.79, *p* = .62; supplementary Table 5). PTSD symptom severity according to total PCL-5 score was not associated with medication adherence and the null association did not vary by intervention condition (*χ*^2^(1) = 2.62, *p* = .11). Including additional clinical covariates provided a better fit to the data than the model excluding them (*χ*^2^(5) = 25.98, *p* < .001; supplementary Table 4), but did not significantly impact findings. A one standard deviation increase in physical comorbidities was associated with significantly increased odds of medication engagement (odds ratio = 1.54; 95% CI, 1.27 to 1.87). No other significant associations were found (Table 2).

### Exploratory Post Hoc Tests

To better understand the results, we examined whether associations between the ISS activation and number of physical comorbidities with psychotherapy engagement and medication adherence varied by intervention group. All interaction effects were non-significant.

## DISCUSSION

This study examined clinical predictors of treatment engagement for complex psychiatric conditions in primary care clinics. To our knowledge, SPIRIT is the largest mental health trial ever conducted in primary care clinics serving rural populations. Our study had minimal exclusion criteria and enrolled a medically complex and socio-demographically diverse sample. Contrary to our hypothesis, clinical characteristics that were expected to negatively impact engagement in pharmacotherapy or psychotherapy did not have stronger effects in the TER arm compared to the TCC arm despite the overall greater level of psychotherapy engagement in the TCC arm. TCC was able to better engage patients in psychotherapy visits via the onsite care manager and sustained proactive outreach. The differences in outreach practices between arms did not impact engagement. Both models can be offered based on primary care practice resource availability. TCC may be more favorable from a population health perspective insofar as it efficiently reaches and links more patients with mental health services compared to TER.

We hypothesized that patients with a higher level of avoidance would be less likely to engage in psychotherapy as indicated in previous studies^23–25^. Avoidance, a core symptom cluster of PTSD, can impair functioning and lead to isolation to escape unpleasant triggering memories^25^. We found no benefit of considering avoidance separately from overall PTSD symptom severity. Neither avoidance nor total PTSD symptom severity was associated with treatment engagement, and thus, our findings suggest neither should be used to drive clinicians’ decisions to initiate treatment for patients with PTSD within integrated or referral telehealth care models.

In exploratory analyses, manic symptom severity according to the ISS activation score was the only negative predictor of psychotherapy initiation. This finding may be in part due to clinician prioritization of treatment embedded in a shared decision-making process. Given the biological underpinning of BD, treatment guidelines for mania and mixed states focus generally on pharmacologic interventions^43,44^ which could result in deferring the initiation of psychotherapy, despite the evidence for effectiveness^45,46^. Lower psychotherapy initiation among those with manic symptoms could also be due to diminished insight, motivation, or level of organization to engage in psychotherapy^44,46^. Our finding calls for intensification of engagement efforts through combined pharmacotherapy and psychosocial interventions for patients in a current manic episode. It also warrants further exploration of clinician prioritization of mental health services for manic patients within specific telehealth care models.

Substance use disorders are highly comorbid with PTSD and BD^10,11^. We found the DAST-10 score was associated with receipt of fewer psychotherapy sessions. Studies conducted with community-based samples^47^ and in addiction programs^48,49^ display a similar trend and point to drug cravings and negative emotions as clinical characteristics associated with disengagement from mental health care. By contrast, alcohol use according to the AUDIT-C did not predict engagement which could be due to primary care-based providers having more experience engaging patients on their alcohol use compared to drug use. Also, the psychotherapy provided in our study did not specifically integrate behavioral approaches to substance use, which is a limitation that future implementation of these telehealth care models should consider addressing.

Individuals with PTSD and mood disorders have a greater likelihood of suffering from the most prevalent physical health conditions such as cardiovascular, endocrine, and pain-related conditions^7,12,50^. This health disparity tends to be greater in rural areas compared to metropolitan sectors ^51^. In this trial, patients had an average of four physical comorbidities including high rates of pain-related illnesses. We found a favorable correlation between the cumulative burden of physical comorbidities and likelihood of psychotherapy and pharmacotherapy engagement. This is consistent with other studies conducted in primary care clinics with patients with PTSD and mood disorders ^50,52,53^. The increased contact with mental health services is critical, particularly for patients with pain-related conditions considering their negative impact on mental health, substance use, and global functioning.

Our results should be appreciated with the following limitations in mind. Firstly, PTSD is highly prevalent in our sample with 92% of those who screened positive for BD also screened positive for PTSD. This is representative of the participating FQHCs in three states. Secondly, psychotherapy was accessed differently in each arm, with TER requiring a referral from a psychiatrist; nevertheless, we did not find any difference between arms with respect to clinical predictors of engagement. Thirdly, we used the psychometric scales administered pre-randomization as predictors of engagement rather than the psychiatric diagnoses assigned by clinicians after randomization to ensure we capture both engaged and non-engaged patients.

## CONCLUSION

Both teleintegrated and telereferral care offer an opportunity to treat patients with chronic and complex psychiatric conditions with otherwise limited access to mental health specialists. Our results suggest mania and substance use, but not PTSD symptom severity, may require additional intervention to support engagement while medical comorbidities may foster care-seeking behaviors. While there was no difference in clinical characteristics predicting engagement between models, onsite care managers engaged patients in more psychotherapy sessions than remote therapists.

## Supplementary Information

ESM 1 (DOCX 37 kb)
