# Supplementary material for: Clinical Predictors of Engagement in Teleintegrated Care and Telereferral Care for Complex Psychiatric Disorders in Primary Care: a Randomized Trial
Source: J Gen Intern Med. 2022 Feb 2;37(13):3361–7. doi: 10.1007/s11606-021-07343-x (PMC9550945; doi:10.1007/s11606-021-07343-x)
Supplement: Supplementary file 1 — (DOCX 37 kb) [file 11606_2021_7343_MOESM1_ESM.docx]

Supplementary Table 3. Physical Health Comorbidities

| **Anemia (%)** |  |
| --- | --- |
| No | 862 (87.0) |
| Yes | 129 (13.0) |
| **Arthritis or any kind of rheumatism. (%)** |  |
| No | 569 (57.4) |
| Yes | 422 (42.6) |
| **Asthma (%)** |  |
| No | 719 (72.0) |
| Yes | 280 (28.0) |
| **Cancer (%)** |  |
| No | 969 (97.6) |
| Yes | 24 (2.4) |
| **Cataracts or other serious vision problem (%)** |  |
| No | 843 (85.1) |
| Yes | 148 (14.9) |
| **Diabetes (%)** |  |
| No | 859 (86.2) |
| Yes | 138 (13.8) |
| **Gall bladder trouble (%)** |  |
| No | 921 (92.7) |
| Yes | 73 (7.3) |
| **Heart disease (%)** |  |
| No | 926 (93.4) |
| Yes | 65 (6.6) |
| **High blood pressure (%)** |  |
| No | 694 (69.7) |
| Yes | 302 (30.3) |
| **Kidney trouble (%)** | |
| No | 922 (92.7) |
| Yes | 73 (7.3) |
| **Lung disease (%)** | |
| No | 926 (93.3) |
| Yes | 66 (6.7) |
| **Migraine headaches (%)** | |
| No | 536 (53.5) |
| Yes | 465 (46.5) |
| **Repeated bladder disorders (%)** | |
| No | 906 (91.1) |
| Yes | 88 (8.9) |
| **Repeated seizures (%)** | |
| No | 944 (94.6) |
| Yes | 54 (5.4) |
| **Repeated stomach problems (%)** | |
| No | 645 (64.6) |
| Yes | 354 (35.4) |
| **Repeated trouble with neck, back, or spine (%)** | |
| No | 383 (38.2) |
| Yes | 619 (61.8) |
| **Stroke (%)** | |
| No | 960 (96.1) |
| Yes | 39 (3.9) |
| **Tuberculosis (%)** | |
| No | 991 (99.1) |
| Yes | 9 (0.9) |
| **Ulcer (%)** | |
| No | 898 (89.9) |
| Yes | 101 (10.1) |
| **Do you have any other medical conditions? (%)** | |
| No | 421 (42.8) |
| Yes | 562 (57.2) |
| *Note.* Answers apply only to people who answered the question, and missing was ranged from 0.3% to 2.1%. | |

Supplementary Table 4A. Zero-inflated Negative Binomial Models for Psychotherapy Engagement (binary)

|  | Model 1 | | Model 2 | | Model 3 | | Model 4 | |
| --- | --- | --- | --- | --- | --- | --- | --- | --- |
| Variable | B (se) | p-value | B (se) | p-value | B (se) | p-value | B (se) | p-value |
|  |  |  |  |  |  |  |  |  |
| PCL-5 Overall | 0.14 (0.08) | .09 | LRT Model 2 vs Model 1 (df = 6)  = 7.65,  *p* = .26 | | 0.12(0.14) | .40 | 0.15 (0.11) | .15 |
| PCL-5: Intrusions | … | … | -0.10  (0.13) | .43 | … | … | LRT Model 4 vs Model 1 (df = 10)  = 51.83,  *p* <.001 | |
| PCL-5: Avoidance | … | … | 0.15  (0.12) | .21 | … | … |  |  |
| PCL-5 Neg. Cog/Mood | … | … | 0.20  (0.13) | .13 | … | … |  |  |
| PCL-5: Hyperarousal | … | … | -0.07  (0.12) | .57 | … | … |  |  |
| SCL-20 | … | … | … | … | … | … | 0.17  (0.11) | .12 |
| ISS activation | … | … | … | … | … | … | -0.36  (0.09) | <.001 |
| DAST-10 | … | … | … | … | … | … | -0.13  (0.08) | .14 |
| AUDIT-C | … | … | … | … | … | … | 0.13  (0.10) | .19 |
| Physical comorbidities | … | … | … | … | … | … | 0.15  (0.09) | .07 |
|  |  |  |  |  |  |  |  |  |
| **Condition X Predictor**  **Interactions (Ref. = TCC)** | |  |  |  | LRT Model 3 vs Model 1 (df = 2)  = 3.03,  *p* = .21 | |  | |
| SCL-20 | … | … | … | … | … | … | … | … |
| ISS activation | … | … | … | … | … | … | … | … |
| PCL-5 Overall | … | … | … | … | 0.05(0.17) | .78 |  | |
| PCL-5: Intrusions | … | … | … | … | … | … | … | … |
| PCL-5: Avoidance | … | … | … | … | … | … | … | … |
| PCL-5 Neg. Cog/Mood | … | … | … | … | … | … | … | … |
| PCL-5: Hyperarousal | … | … | … | … | … | … | … | … |
| DAST-10 | … | … | … | … | … | … | … | … |
| AUDIT-C | … | … | … | … | … | … | … | … |
| Physical comorbidities | … | … | … | … | … | … | … | … |

*Note.* Non-engagement is specified as the reference group, meaning that positive effects are predictive of initiating psychotherapy.

Supplementary Table 4B. Zero-inflated Negative Binomial Models for Psychotherapy Engagement (count)

|  | Model 1 | | | | Model 2 | | | Model 3 | | | Model 4 | | | |
| --- | --- | --- | --- | --- | --- | --- | --- | --- | --- | --- | --- | --- | --- | --- |
| Variable | B (se) | | p-value | | B (se) | p-value | | B (se) | p-value | | B (se) | p-value | | |
|  |  | |  | |  |  | |  |  | |  |  | | |
|  |  | |  | |  |  | |  |  | |  |  | | |
| PCL-5 Overall | 0.02  (0.04) | | .53 | | LRT Model 2 vs Model 1 (df = 6)  = 7.65,  *p* = .26 | | | 0.07  (0.05) | | .13 | 0.052 (0.04) | | .24 | |
| PCL-5: Intrusions | … | | … | | 0.13 (0.06) | .03 | | … | … | | LRT Model 4 vs Model 1 (df = 10)  = 51.83,  *p* <.001 | | | |
| PCL-5: Avoidance | … | | … | | -0.01 (0.05) | .83 | | … | … | |  |  |  |  |
| PCL-5 Neg. Cog/Mood | … | | … | | -0.05 (0.06) | .42 | | … | … | |  |  |  |  |
| PCL-5: Hyperarousal | … | | … | | -0.04 (0.06) | .53 | | … | … | |  |  |  |  |
| SCL-20 | … | | … | | … | … | | … | … | | -0.02 (0.05) | .69 | | |
| ISS activation | … | | … | | … | … | | … | … | | -0.05 (0.04) | .19 | | |
| DAST-10 | … | | … | | … | … | | … | … | | -0.13 (0.04) | .002 | | |
| AUDIT-C | … | | … | | … | … | | … | … | | -0.01 (0.04) | .73 | | |
| Physical comorbidities | … | | … | | … | … | | … | … | | 0.10  (0.04) | .009 | | |
|  |  | |  | |  |  | |  |  | |  |  | | |
| **Condition X Predictor**  **Interactions (Ref. = TCC)** | | | |  |  | |  | LRT Model 3 vs Model 1 (df = 2)  = 3.03,  *p* = .21 | | |  | | | |
| SCL-20 | | … | | … | … | | … | … | | … | … | | | … |
| ISS activation | | … | | … | … | | … | … | | … | … | | | … |
| PCL-5 Overall | | … | | … | … | | … | -0.14  (0.08) | | .08 |  | | | |
| PCL-5: Intrusions | | … | | … | … | | … | … | | … | … | | | … |
| PCL-5: Avoidance | | … | | … | … | | … | … | | … | … | | | … |
| PCL-5 Neg. Cog/Mood | | … | | … | … | | … | … | | … | … | | | … |
| PCL-5: Hyperarousal | | … | | … | … | | … | … | | … | … | | | … |
| DAST-10 | | … | | … | … | | … | … | | … | … | | | … |
| AUDIT-C | | … | | … | … | | … | … | | … | … | | | … |
| Physical comorbidities | | … | | … | … | | … | … | | … | … | | | … |

Supplementary Table 5. Prediction Models for Pharmacotherapy (binary)

|  | Model 1 | | Model 2 | | Model 3 | | | Model 4 | |
| --- | --- | --- | --- | --- | --- | --- | --- | --- | --- |
| Variable | B (se) | p-value | B (se) | p-value | B (se) | p-value | | B (se) | p-value |
|  |  |  |  |  |  |  | |  |  |
| PCL-5 Overall | -0.14 (0.09) | .11 | LRT (df = 3)  = 1.79,  *p* = .62 | | 0.00 (0.12) | | .98 | -0.22 (0.12) | .95 |
| PCL-5: Intrusions | … | … | 0.02 (0.13) | .91 | … | … | | LRT Model 4 vs Model 1 (df = 5)  = 25.98,  *p* <.001 | |
| PCL-5: Avoidance | … | … | 0.06 (0.12) | .61 | … | … | |  |  |
| PCL-5 Neg. Cog/Mood | … | … | -0.21 (0.14) | .13 | … | … | |  |  |
| PCL-5: Hyperarousal | … | … | -0.012 (0.13) | .93 | … | … | |  |  |
| SCL-20 | … | … | … | … | … | … | | 0.10 (0.12) | .38 |
| ISS activation | … | … | … | … | … | … | | -0.07 (0.1) | .45 |
| DAST-10 | … | … | … | … | … | … | | -0.11 (0.1) | .27 |
| AUDIT-C | … | … | … | … | … | … | | -0.04 (0.1) | .64 |
| Physical comorbidities | … | … | … | … | … | … | | 0.43 (0.1) | <.001 |
|  |  |  |  |  |  |  | |  |  |
| **Condition X Predictor**  **Interactions (Ref. = TCC)** | |  |  |  | LRT Model 3 vs Model 1 (df = 1)  = 2.62,  *p* = .11 | | |  | |
| SCL-20 | … | … | … | … | … | … | | … | … |
| ISS activation | … | … | … | … | … | … | | … | … |
| PCL-5 Overall | … | … | … | … | -0.28 (0.18) | .11 | |  | |
| PCL-5: Intrusions | … | … | … | … | … | … | | … | … |
| PCL-5: Avoidance | … | … | … | … | … | … | | … | … |
| PCL-5 Neg. Cog/Mood | … | … | … | … | … | … | | … | … |
| PCL-5: Hyperarousal | … | … | … | … | … | … | | … | … |
| DAST-10 | … | … | … | … | … | … | | … | … |
| AUDIT-C | … | … | … | … | … | … | | … | … |
| Physical comorbidities | … | … | … | … | … | … | | … | … |
